# Sick sinus syndrome diagnosed after a sinus arrest during treatment for zygomatic fracture: a case report

**DOI:** 10.1186/s12903-023-03413-0

**Published:** 2023-09-19

**Authors:** Hiroki Hayashi, Atsushi Abe, Tetsushi Oguma, Yu Ito, Atsushi Nakayama

**Affiliations:** 1 Department of Oral and Maxillofacial Surgery, Nagoya Ekisaikai Hospital, Nagoya, Japan; 2 Department of Oral and Maxillofacial Surgery, School of Dentistry, Aichi Gakuin University, Nagoya, Japan

**Keywords:** Sick sinus syndrome, Bradycardia-tachycardia syndrome, Sinus arrest, Syncope, Zygomatic fracture

## Abstract

**Background:**

Intraoperative sinus arrest is rarely seen during zygomatic fracture treatment. The patient was diagnosed with sick sinus syndrome based on repeated postoperative sinus arrest, which could have resulted in death if diagnosed late, making this case very significant to report.

**Case presentation:**

Sick sinus syndrome is an arrhythmia associated with reduced automaticity of the sinoatrial node or impaired sinoatrial node conduction. We report the case of a 67-year-old man diagnosed with the syndrome after a sinus arrest that occurred during a zygomatic fracture treatment. The patient had cheek pain and mouth opening disorder, dizziness after fainting and sustaining a facial injury. Preoperative examination determined that the syncope was due to drug-induced arrhythmia, and surgery was authorized after drug withdrawal. During the operation, sinus arrest was observed due to trigeminal vagal reflex, and heart rate was restarted by stopping the operation and chest compressions. After the surgery, the patient showed symptoms of dizziness and palpitations, and sinus arrest following atrial fibrillation and supraventricular tachycardia, which was diagnosed as sick sinus syndrome, and a pacemaker was implanted. Currently, 8 years have passed since the surgery, and there are no symptoms of mouth opening disorder, dizziness, or palpitations.

**Conclusions:**

In the case of maxillofacial injuries due to syncope, cardiogenic syncope is a possibility, and repeated syncope is a risk for death due to delayed diagnosis. There are no reports of maxillofacial trauma leading to a diagnosis of sick sinus syndrome. The purpose of this case report is to disseminate the importance of diagnosing the cause of syncope as well as injury treatment.

## Background

Sick sinus syndrome is an arrhythmia caused by damage to the sinus node and surrounding tissues and is associated with reduced automaticity of the sinus node or sinus node conduction defects [1]. The etiologic factors are mainly divided into intrinsic and extrinsic causes. Intrinsic causes include abnormalities in fibrosis, ion channel formation, and sinus node function, while extrinsic causes include drugs, metabolism [2]. The incidence increases with age and is more common in the elderly [3]. In the United States, the incidence has been increasing since 2012, with more than 75,000 cases each year [4]. The incidence of sick sinus syndrome is estimated to be 0.08%. The prognosis is good with treatment intervention, as it rarely causes sudden death, but the mortality rate in untreated patients is 2% [5, 6]. The patients are prone to syncope and dizziness due to inability to maintain sufficient cardiac output caused by atrioventricular nodal conduction defects and paroxysmal supraventricular tachycardia. Although a Holter electrocardiogram is useful to make a definitive diagnosis, it is difficult to distinguish it from other similar diseases [7, 8] and it may be diagnosed as sick sinus syndrome based on arrest or arrhythmia. This report aims to describe a case of repeated sinus arrest during hospitalization for a zygomatic fracture, which was diagnosed as sick sinus syndrome.

## Case presentation

### Medical history and medication

The patient was a 67-year-old Japanese man, with hypertension, paroxysmal atrial fibrillation, diabetes mellitus, liver dysfunction, hyperlipidemia and was on medication with vildagliptin, voglibose, ursodeoxycholic acid, pravastatin sodium, β-blocker, digoxin, febuxostat. There was no previous history of surgery or syncope episodes, and the patient had been asymptomatic for illnesses including paroxysmal atrial fibrillation. Paroxysmal atrial fibrillation was not noted on scheduled examination or laboratory tests.

### History of present illness

In July 2014, while getting out of his car, he lost consciousness, fell down, and injured the left side of his face. He regained consciousness quickly, had examination at neurology department after he was found to have pain in the left side of his cheek, difficulty in opening his mouth, and dizziness. A close examination of imaging revealed a fracture of the zygomatic bone, and the patient was referred to our department for an initial consultation on the same day.

### Examination

**Fig. 1:**
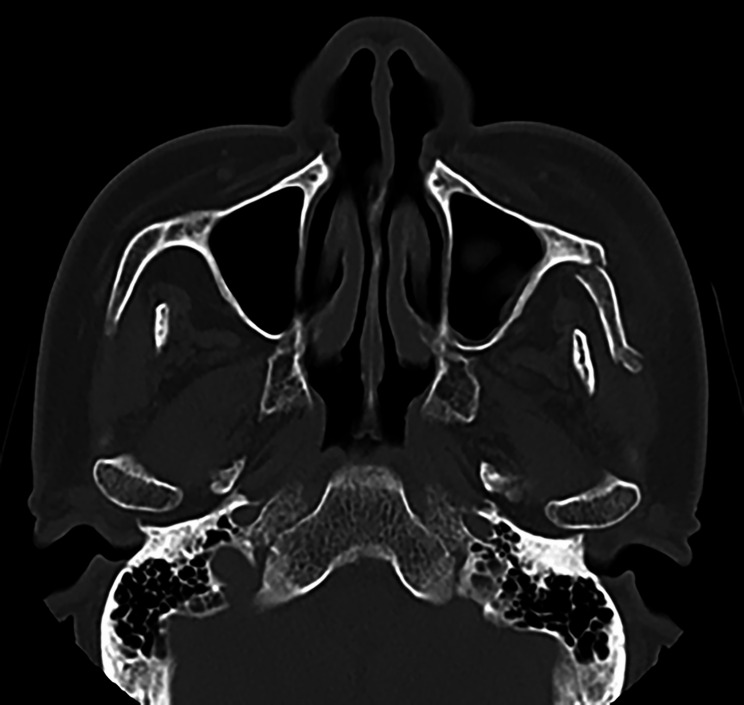
CT image at initial examination. The left zygomatic maxillary suture was fractured, and zygomatic arch was depressed. Zygomatic bone was displaced medially and interfered with the left muscular process

**Fig. 2:**
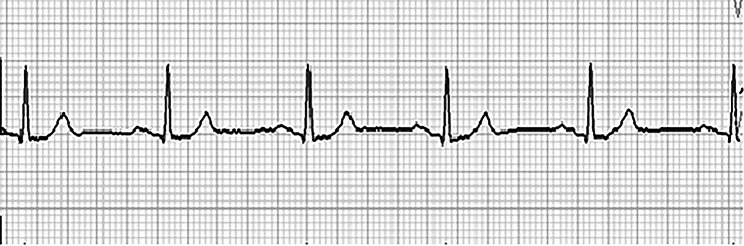
ECG at initial examination. Normal sinus rhythm was shown

**Fig. 3:**
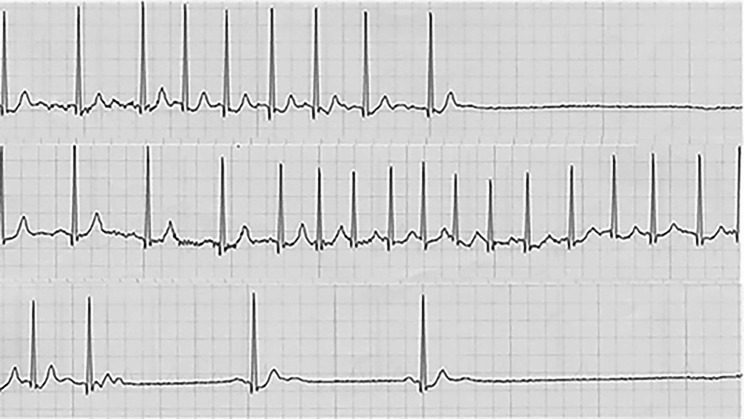
Holter ECG at initial examination. Tachycardic atrial fibrillation and sinus arrest were found

At initial examination, he was conscious and able to walk independently. He did not have chest pain or dyspnea,　complained of dizziness and palpitations. Extraoral examination revealed diffuse swelling and subcutaneous hemorrhage in the left buccal region. Dysesthesia of the left suborbital nerve area was observed. There was no left-sided ocular injury, no diplopia, and no loss of visual acuity. Intraoral examination revealed no damage to the teeth was observed. The denture was damaged as a result of the injury, and no occlusal abnormality was found. The amount of opening was 18 mm (the distance between the upper and lower mandibular crests at the maximum opening). Computed tomography (CT) revealed fracture line was observed at the left zygomatic maxillary suture, the left zygomatic arch was depressed, and the zygomatic bone was displaced medially and interfered with the left muscular process (Fig. 1). Chest X-ray showed no abnormalities. A 12-lead electrocardiogram showed a heart rate of 63 beats per minute with no bradycardia and normal sinus rhythm (Fig. 2). Echocardiography revealed moderate aortic regurgitation, mild mitral, tricuspid, and pulmonary regurgitation, and mild left atrial enlargement, but left ventricular ejection fraction was 65.1% and no abnormalities in cardiac function were observed. Preoperative head CT and electroencephalography revealed no evidence of central nervous system disease. Holter electrocardiogram showed tachycardic atrial fibrillation and sinus arrest consistent with vertigo symptoms (Fig. 3). A clinical diagnosis was zygomatic fracture. The attending cardiologist at our hospital concluded that the patient was taking beta-blockers and digoxin, which may have inhibited the function of the sinoatrial node and induced syncope. He indicated that surgery could be performed under drug withdrawal, and since we found that the patient had difficulty in opening his mouth due to dislocation of the zygomatic bone, we scheduled a zygomatic fracture contemplative repair and fixation procedure under general anesthesia.

### Treatment

**Fig. 4:**
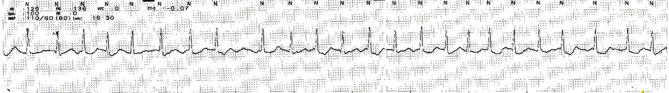
ECG during operation. Initially, sinus rhythm was found, but a 20-second sinus arrest was observed. After chest compressions, it changed into sinus rhythm

**Fig. 5:**
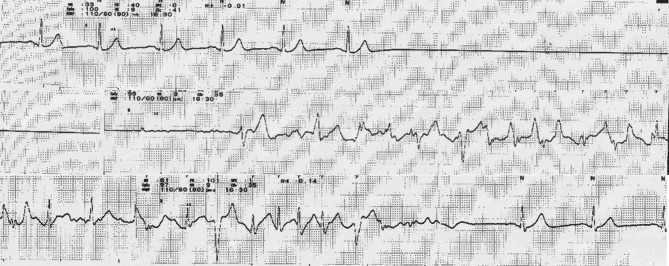
ECG at extubation. Tachycardic atrial fibrillation was observed. Sinus arrest was not found

**Fig. 6:**
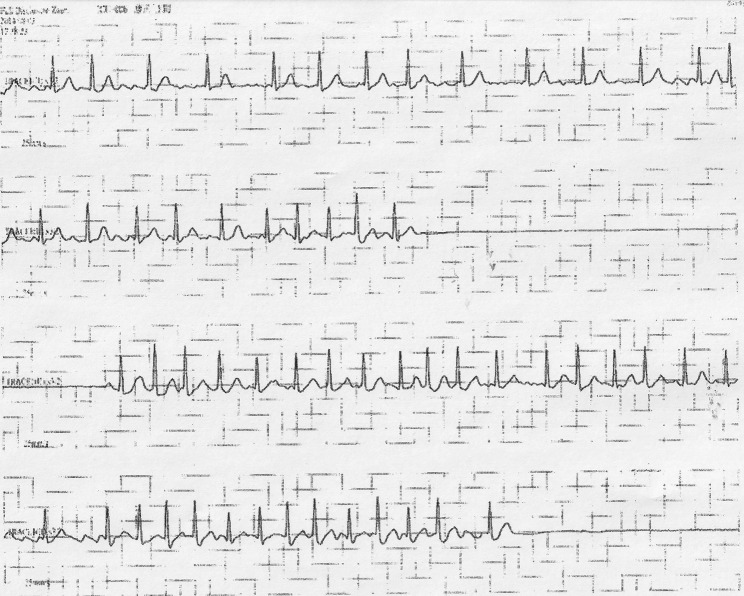
ECG after operation. Tachycardic atrial fibrillation and supraventricular tachycardia followed by repeated sinus arrest were found

Isozol (100 mg) was used as the induction drug for general anesthesia, and sevoflurane (17.6 ml), fentanyl (0.1 mg), remifentanil 0.1 γ (4.2 ml/h) and Eslax (40 mg) were used as maintenance drugs. During induction of anesthesia, there was no abnormality in vital signs and no abnormal waveforms in electrocardiogram. The patient was repositioned and immobilized using the intraoral approach according to the usual technique. The gingiva was incised through the oral vestibule, and the mucoperiosteal valve was detached and elevated to expose the fracture line. Until this point, the electrocardiogram was in normal sinus rhythm. However, when the buccal elevator was used for the preparation of the bone fragment, sinus arrest continued for 20 s, and the systolic blood pressure dropped to 30 mmHg. The surgery was immediately interrupted and chest compressions were started, and the patient achieved normal sinus rhythm without drug administration or defibrillation (Fig. 4). The surgery was resumed and completed without any sinus arrest or ECG abnormality. Although no sinus arrest was observed during extubation, tachycardic atrial fibrillation was observed (Fig. 5). The patient was managed in the intensive care unit (ICU) in the postoperative period. From the day of surgery to the third day, atrial fibrillation and supraventricular tachycardia followed by sinus arrest were observed (Fig. 6). Just before the arrhythmia occurred, dizziness and subsequent syncope were observed. Although the patient had stopped taking beta-blockers and digoxin before the surgery, the diagnosis of sick sinus syndrome (bradycardia-tachycardia syndrome) was made instead of drug-induced arrhythmia because of the abnormal electrocardiogram.

The patient was diagnosed as a candidate for pacemaker implantation due to the continued presence of postoperative arrhythmia, and pacemaker implantation was performed on the third postoperative day. After the pacemaker implantation, symptoms of dizziness and palpitations disappeared, and he discharged on the 13th postoperative day. The patient was followed up for 8 years with no symptoms of mouth opening disorder, dizziness, or palpitations.

## Discussion

Sick sinus syndrome is caused by an abnormality in the automaticity of the sinus node itself or a conduction defect from the sinus node to the atria, resulting in bradycardia and associated symptoms [5]. Clinical symptoms include shortness of breath, fatigue on exertion, lightheadedness, dizziness, and syncope, and the diagnosis is made by electrocardiography and electrophysiological examination. Based on the characteristics of the electrocardiographic waveform, this disease is classified into three types using the Rubenstein classification: type I is sinus bradycardia, type II is sinus arrest and sinus atrial block, and type III is bradycardia-tachycardia syndrome [9]. In this case of bradycardia-tachycardia syndrome, bradyarrhythmia (sinus bradycardia, sinus atrial block, sinus arrest) and paroxysmal tachycardia (atrial fibrillation, supraventricular tachycardia, ventricular tachycardia) coexist, causing syncope and heart failure, about 80% of which are idiopathic [10], and treatment methods include drug therapy and pacemaker therapy, although asymptomatic patients are not usually treated with such therapy. In some cases, patients may be followed up. The frequency of sudden death with pacemaker therapy is 0.06%, while the frequency of sudden death without therapy is estimated to be 2% [6]. In the present case, there were no abnormalities on history taking, 12-lead electrocardiogram, blood test, head CT, and EEG. Echocardiography showed valvular disease, but no abnormality that would cause syncope. On the other hand, Holter electrocardiography showed sinus arrest after atrial fibrillation. Since the patient was taking beta-blockers and digitalis preparations that suppress sinus node function, the cause of syncope was diagnosed as drug-induced sinus arrest, and surgery was performed after drug administration was discontinued.

This is the first report of an injury limited to the maxillofacial region leading to a diagnosis of sick sinus syndrome. The report of a patient diagnosed with sick sinus syndrome due to a head injury caused by syncope was accepted, and the commonality with this case was a history of atrial fibrillation [11]. Interestingly, atrial fibrillation and sick sinus syndrome have been reported to interact [12], and therefore, when there is a history of atrial fibrillation, sick sinus syndrome should be strongly suspected as the cause of syncope. It is often the case that latent sick sinus syndrome that could not be diagnosed before surgery is diagnosed after surgery and anesthesia, as in the present case, and there have been reports of bradycardia and sinus node arrest during anesthesia induction and intraoperative pacing [10, 13]. During the perioperative period, the use of anesthetics tends to cause cardiac instability, and bradycardia or sinus arrest can result in compensatory changes such as increased preload and increased cardiac output per cycle. But, there are cases in which compensatory changes are not feasible, and failure to maintain sufficient cardiac output may result in lethal arrhythmias. Factors that induce intraoperative arrhythmias include hypoxemia, hypercarbia, shallow anesthesia, vagal reflex, air embolization, acidosis, alkalosis, and drug use (beta-blockers, digoxin, anesthetics). In this case, there were no abnormalities in vital signs, ventilatory status, or electroencephalographic monitoring during anesthesia induction, endotracheal intubation, and general anesthesia. Beta-blockers and digoxin were withdrawn preoperatively and were not used intraoperatively. Therefore, factors such as hypoxemia, hypercarbia, shallow anesthesia, and drug use were ruled out. On the other hand, no arrhythmia was observed before sinus arrest, and sinus arrest occurred immediately after the repair of the zygomatic fracture, and the patient became in sinus rhythm when the surgery was terminated, so the possibility that the trigeminal vagal reflex was activated by stimulation from the bone fragment cannot be denied [14–16]. The trigeminal reflex during oral surgery is thought to be caused by stimulation of the second and third branches of the trigeminal nerve, and there have been reports of the trigeminal vagus reflex during contemplative fixation of zygomatic fractures. In fact, the patient reported a sinus arrest during the repair of a zygomatic fracture fragment, which suggests that the reflex was caused by the same mechanism as in the present case [15–18]. Cha et al. [19] reported that the presence of preexisting cardiac disease predisposes to the trigeminal vagus reflex. Therefore, the present patient had an underlying sick sinus syndrome, which may have been related to the intraoperative trigeminal vagus nerve reflex induction. After the surgery, tachycardic atrial fibrillation was observed during extubation, and although sinus arrest was not observed, atrial tachyarrhythmia with dizziness, palpitations, and other symptoms, and subsequent sinus arrest were observed. Although the diagnosis of drug-induced arrhythmia was made during the preoperative examination, similar symptoms and electrocardiographic findings were repeatedly observed even after discontinuation of drug therapy. Based on these findings, the intraoperative sinus arrest was diagnosed as trigeminal vagal reflex, and the postoperative sinus arrest was diagnosed as sinus arrest due to sick sinus syndrome (bradycardia-tachycardia syndrome), not drug-induced arrhythmia, and a pacemaker was inserted.

In the treatment of traumatic injuries in the maxillofacial region, undiagnosed lesions are often present and are more likely to be missed if urgent surgery is required [20]. This requires careful interviewing and examination. It is also necessary to consider the timing of surgery when the underlying disease is present. In the present case, an opening disturbance was observed at the time of initial examination, and surgery was performed on the 5th day after the injury. However, there was no persistent bleeding or significant pain, and if surgery is indicated when the patient is repaired within 2 weeks, which is　the time of fresh fracture, it was considered necessary to stop drug administration and follow-up, and to consider giving priority to identifying the cause of the dizziness and palpitations. In Japan, where the elderly population is rapidly increasing, the number of elderly patients with multiple systemic diseases and taking multiple medications is increasing, and in the case of facial fracture after syncope, the cause of syncope should be identified as much as possible before surgery. For differential diagnosis of syncope, history taking, blood tests, electrocardiography, echocardiography, echocardiography, and central nervous system examination should be performed to consider sudden changes due to drug interactions, potential diseases [21]. In the case of sinus arrest during emergency surgery, intraoperative transvenous cardiac pacing was inserted, or transcutaneous pacing was prepared after resuming heartbeat by chest compression and dopamine administration, and the importance of consultation with cardiology and anesthesiology was reaffirmed.

## Conclusion

In the treatment of maxillofacial injuries, the urgency of the situation may lead to surgical intervention in some cases prior to investigation of the cause of the injury. The patient had no history of syncope, was diagnosed with medication induced syncope, withdrew from the medication, and subsequently had no syncope by the time of surgery. If the follow-up period before surgery had been longer, syncope could have occurred again and the diagnosis could have been made at that time. If cardiogenic syncope remains undiagnosed and untreated, it may cause death or new trauma due to repeated syncope, making diagnosis of the cause of syncope extremely important.

## Data Availability

The datasets used and/or analyzed during the current study are available from the corresponding author on reasonable request.

## List of abbreviations

CT: Computed tomography
ICU: Intensive care unit
